# Aggressive behavior in adolescent: The importance of biopsychosocial predictors among secondary school students

**DOI:** 10.3389/fpubh.2023.992159

**Published:** 2023-04-14

**Authors:** Fatimah Ahmad Fauzi, Nor Afiah Mohd Zulkefli, Anisah Baharom

**Affiliations:** Department of Community Health, Faculty of Medicine and Health Sciences, Universiti Putra Malaysia, Selangor, Malaysia

**Keywords:** adolescent, aggression, school students, biopsychosocial, predictors

## Abstract

**Introduction:**

Overt aggression is a common type of aggression observed among adolescents, which is apparent and outward confrontational acts manifested physically and verbally, such as fighting and shouting. It has become a major public health concern, as it results in detrimental health impacts like injury, mental health, and social problems.

**Methods:**

An observational study was conducted among 16-year-old school students to determine their biopsychosocial predictors, using stratified proportionate population sampling. Pre-tested surveys were distributed to measure students’ aggression, biological, psychological, and social factors.

**Results:**

A total of 463 students from four public secondary schools participated in the study, with a median aggression score was 23.00 (IQR=12.00). The significant predictors of aggression from multivariate analysis were Malay race, frequent dessert intakes, attitude towards aggression, low family income, and peer deviant affiliation (*F* [8, 244] = 15.980, *p* < 0.001, adjusted *R*^2^ = 0.290).

**Discussion:**

Adolescent aggression determinants are collectively impacted as a result of biological, psychological, and social predictors and need to be focused on in interventional strategies.

## Introduction

Aggressive behavior and its associated psychiatric disorders can occur throughout a person’s life span, from childhood to adolescents, adulthood, and the elderly (1). Aggression is defined as any action with the intention to cause harm, injury, or pain toward another person (2). This includes overt and covert aggression, which are the most common types of aggression that have been examined among children and adolescents. The apparent and outward confrontational acts of overt aggression can be manifested in both physical and verbal aggression, such as fighting and shouting (3), while covert aggression in social relationships is more secretive and manipulative, by way of isolation and spreading of rumors (4). Aggression and violence are leading causes of death in older male adolescents that can deter their ability to grow and develop optimally, as their current and future health is being jeopardized (5). If these habits remain untreated, they may progress to more detrimental psychological problems including anxiety and depression (6). Therefore, it would be worthwhile to identify and understand the factors that underpin their misconduct and to identify effective interventions for aggressive behavior development among adolescents.

Adolescent health is important in every nation for them to reach adulthood with both good mental and physical well-being, thereby protecting the health of future generations. It is considered to be a unique phase of human development, which can be stressful and influenced by specific and different types of behavior including daring, distraction, and rebellion (7). Aggressive behavior in adolescents can also manifest in their risk-taking behavior, which has been a public health concern as it may be linked to injury and personal trauma (5). In Malaysia, the involvement of school children and adolescents is high in juvenile cases, such as robbery, rape, criminal threatening, and rioting (8). According to the Global School Health Survey (GSHS) conducted among secondary school students in Malaysia in 2012 (9), over a fourth (25.9%) of them were involved in physical fights. The highest percentage was apparent in students aged 16–17 years old (32.6%) who were seriously injured one or more times over the past 12 months. The prevalence was higher when compared to neighboring countries in the Western Pacific Region such as Brunei (23.4%) and Cambodia (10%).

Biopsychosocial risk factors play a vital role in the etiology of aggressive behaviors, which can form the basis for a biopsychosocial approach to prevent and provide intervention for all problems relating to adolescent aggression (10). The conundrum of an adolescent behavioral problem can be explained by an integrated approach of the biopsychosocial model, which incorporates interactive and multiplicative processes of biopsychosocial factors (11).

A systematic review of psychosocial predictors of adolescent aggression (12) showed that personality traits, emotional problems, peer influence, and substance use were among the highest reported predictors. In local observational research among school students, psychosocial factors of attitude toward aggression (13), antisocial personality (13), family environment (14), and teacher and peer attachment (13) have shown significant associations with adolescent aggression. However, their interrelation with significant internal stimuli of biological aspects like race (15), poor nutrition, and low-quality diet (16) have not yet been investigated in determining the affected adolescent intervention requirements. Some of the local research on adolescent aggression also does not apply behavioral theory, which is crucial in behavior-related medical research (17).

The study aims to determine the biopsychosocial predictors of aggressive behavior among secondary school students in a district experiencing a high number of disciplinary problems and crime juvenile index cases in the state. Although some international prevention programmes have shown to be successful (11), their application in the local context may vary as the Malaysian education system and cultural beliefs differ from Western countries. Therefore, a more targeted approach needs to be designed for a more suitable intervention programme, taking into consideration important biopsychosocial predictors.

## Materials and methods

This is a cross-sectional study among 484 secondary school students, aged 16 years old, in selected public secondary schools in Hulu Langat, Selangor, Malaysia. An ethics approval application was made as the study involved human subjects. This involved obtaining three levels of permissions from (1) campus-based institutional review boards by the “Ethics Committee for Research Involving Human Subject of Universiti Putra Malaysia” (JKEUPM Ref No: JKEUPM-2018-228), (2) organizations in charge of the school, and (3) individuals providing the data. Parents’/guardians’ consents were obtained before the students’ involvement as they were underage. Assent for study participation from the students was also obtained so that they thoroughly understand their responses’ confidentiality and anonymity.

### Sample

School students aged 16 years old were chosen to represent adolescents in this study based on previous evidence that showed being older (including 16-year-olds) was associated with moderate aggressive behavior (15). The highest number of crime involvement among school students was also reported among those aged 16–18 years old based on the Royal Malaysian Police data (18). In public secondary schools, students taking part in a final exam year, those aged 17 years old, were not allowed to be included in a research study. Thus, only 16-year-old students were included in this study. Illiterate students and those with learning disabilities were excluded from this study, as they may experience difficulties in answering the self-administered questionnaire. The sample size was calculated using the formula for multivariate linear regression (19) with adjustment for an estimated non-response rate of 30%, non-eligible rate of 10%, attrition rate of 32.8% (20), and design effect (21). This resulted in a final sample size of 484.

A stratified probability proportional to size (PPS) sampling method was applied in this study, from estimated 12,012 secondary school students in 36 Public secondary schools in Hulu Langat district. The list of school in the primary sampling frame was arranged descending according to the size of the school (number of 16-year-old students) from highest 648 to the smallest 111. The number of schools to be sampled were decided based on the sample size divided by the size of the smallest school (484/111 = 4.4 ≈ 4). The final four schools were randomly sampled using systematic sampling with equal groups of 121 students sampled from each selected school (484/4 = 121).

### Measurements

The data set was collected *via* self-administered questionnaires that were distributed and collected by the researcher without involving the school teachers or staff. The selected and consented respondents answered the questionnaires in their respective schools after written consent was obtained from their parents or guardians. As part of quality control, the content and face validity of the study instrument was performed to assess its concept representativeness and comprehensibility among similar populations. The content of the questionnaire was validated by a preliminary discussion with five research experts among Public Health physicians, educational lecturers, and psychologists using Item-Content Validity Index (I-CVI). Questionnaire items that were agreed upon by all experts (CVI = 1) were maintained and translated before being further pre-tested. The WHO (22) translation and adaptation of instruments’ process was applied as follows: forward translation, expert panel, back-translation, pre-testing, and final version (22). As for face validity, it was performed by interviewing several students who were not included in the study to evaluate the questionnaire items’ phrasing and comprehensibility. Relevant comments and suggestions were applied for questionnaire amendments. Upon pre-testing, the revised questionnaire was retested and showed acceptable reliability with all adjusted constructs, Cronbach’s Alpha value of 0.70, and more (23).

The psychometric properties of the questionnaire were also assessed by performing an Exploratory Factor Analysis (EFA) on several validated scales used in the questionnaire. The purpose of this analysis was to test the underlying factor structures among several variables, as it analyses the shared variance of certain items in the questionnaire. A total number of 154 respondents were recruited in the pilot study to fulfill the subject-to-item ratios of 10:1 and 5:1 (24). Several items with factor loading <0.32 (25) were removed from the original scale. After a retest on the adjusted scale was performed, scales that measured psychopathic traits and domestic violence exposure showed similar factor solutions to the original versions with a total of 35.19 and 46.84% of the variance, respectively. Other scales’ factor structures were adjusted accordingly based on these findings.

### Dependent variable: Aggressive behavior

The aggression scale was adapted to measure aggressive behavior in this study. It was originally developed by Orpinas and Frankowski (26), measuring the most common overt aggression of physical aggression (pushing, slapping, kicking, hitting), verbal aggression (encouraging other students to fight, threatening to hit or hurt, teasing, name-calling) and anger (getting angry easily, being angry most of the day) (26). The students were asked to rate their behavior frequency, ranging from 1 *(0 times)* to 7 *(6 or more times)* in the past 7 days to minimize recall bias. Final total scores were calculated for each respondent and scores ranged from a minimum of 13 to a maximum of 91. The reliability of the instrument was good with Cronbach’s Alpha value of 0.83.

### Independent variables

#### Biological factors

Sex was self-reported as either male or female. Race was divided into the following four main groups, Malay, Chinese, Indian, and others. A head injury history was obtained from a single question regarding previous head injuries sustained by students that required medical attention or resulted in unconsciousness (27). The internal stimuli of the biological cause of nutritional deficiency have been shown by many researchers in predisposing aggression (1, 16). The nutritional deficiency was assessed by taking into account food insufficiency, junk food, and low produce intake (16). Food insufficiency was determined based on the occurrence of the students’ experience of hunger due to insufficient food availability at home from *never* (0) to *very often* (3). Junk food consumption was assessed on their weekly frequency intake of sugary drinks, fast food, and desserts. The response ranged from *never* (0) to *every day or more than once per day* (7). To ascertain low produce consumption, they were asked questions relating to the weekly frequency of fruit and vegetable consumption, resulting in similar responses.

#### Psychological factors

Attitude toward aggression questions were derived from six self-report items developed by Bosworth and Espelage in 1995 (28). This measures the acceptability of aggressive behavior, particularly fighting, with four choices of responses: (1) *strongly agree*, (2) *agree*, (3) *disagree*, and (4) *strongly disagree*. Higher total scores signify a positive attitude toward aggression. Normative beliefs regarding aggression questions were based on 12 possible appropriate reactions in a confrontational situation with a peer who had provoked the opponent by using a 4-point scale from 0 *(not at all ok)* to 3 *(totally ok)*, that was developed by Möller and Krahé (29). Higher scores indicate a higher acceptance of aggressive responses. Psychopathic personality traits of callous-unemotional, grandiose-manipulative, and impulsive-irresponsible were investigated by using the 12 items of the Youth Psychopathic Inventory Short Form (YPI-S) developed by van Baardewijk et al. (30). The items were rated on a four-point Likert scale response from 1 *(does not apply at all)* to 4 *(applies very well)*. The total scores ranged from 12 to 48, with higher scores indicative of increased psychopathy personality traits. For emotional intelligence, 23 items of the Schutte Self-Report Emotional Intelligence Test (SSEIT) were adapted (31), which included four dimensions of emotional intelligence: Optimism, Social Skills, and Utilization of Emotions. The reliability of the instruments was good with Cronbach’s Alpha value of 0.88 for normative beliefs regarding aggression, 0.81 for personality traits, and 0.92 for emotional intelligence measurements.

#### Social factors

Family income was self-reported based on the total household monthly income. Family environment investigation covered both family relationship and family personal growth dimensions. The 18 items of the Brief Family Relationship Scale (32) measured the students’ perception of their family relationship functionality quality, their sense of belonging to the family, the open expression between family members, and family conflictual interactions (reversely scored). As for single-parent status, the questions were asked based on the current number of parents who live with and take care of the students. Peer deviant affiliation questions were adapted from the National Youth Survey (33). A final 10 items were included that measured the close friends’ deviant activities over the past 6 months such as cheating, destruction of property, and rule breaking. Questions regarding domestic violence were adapted from the Children’s Exposure to Domestic Violence (CEDV) scale, which included 12 items of violence and other victimization (34) Questions regarding smoking, alcohol consumption, and substance abuse were adapted from the Adolescents Health Survey 2017 of National Health Morbidity Survey 2017 (35). The reliability of the instruments was good with Cronbach’s Alpha value of 0.82 for family environment, 0.79 for peer deviant affiliation, and 0.86 for domestic violence measurement.

### Analytic strategy

The descriptive analysis for continuous variables comprised percentage, mean, and median. Bivariate analysis of simple correlation analysis and simple linear regression tests were used to measure the correlations and linear relationships between independent variables and dependent variables. To determine the biopsychosocial predictors of adolescent aggression, a standard multivariate linear regression test was carried out and the model with the highest variance explanation (*r*^2^) and number of predictors was chosen as the final model. The assumptions that were fulfilled include adequate sample size, no multicollinearity, singularity, outlier adjustment, and checking for normality, linearity, homoscedasticity, and independence of residuals (36). The level of significance α was set at 0.05 with a 95% Confidence Interval, not including one.

## Results

From a total of 484 eligible respondents who were approached for this study, 463 gave consent and answered the questionnaire. This resulted in a response rate of 95.7%. The data set was cleaned and checked for outliers and missing data. There was no variable with more than 5% missing values and cases were excluded only if data required for specific analysis were missing. Outliers were identified and automatically removed after verification with the questionnaire (36). A normality test on numerical data showed normal distribution except for aggressive behavior, household monthly income, domestic violence, and peer deviant affiliation, which were all positively skewed to the left. They were mathematically transformed based on the total scores’ distribution using a square root and logarithm transformations for later parametric statistical tests execution.

### Descriptive analysis

The median total score of aggression obtained from secondary school students in Hulu Langat was 23 (IQR: 12) with a minimum score of 13 and a maximum score of 91. The median frequency of the students acting aggressively, such as fighting when someone hit him/her, teasing other students to make them angry, easily angered, and making fun of other students in the previous 1-week period, was double in the previous week. Other acts of aggression such as threatening, calling names, and physical fighting were reported only once in the previous week. The distribution of sex in the study is almost equal with 219 (47.3%) male and 244 (52.7%) female respondents. The race distribution was mainly Malay (75.4%), followed by Chinese (17.1%), Indian (5.2%), and other races (2.4%) including Orang Asli, Iban, Kadazan, and Murut.

### Bivariate analysis

As shown in Table 1, there was a significant linear relationship between the male sex (*β* = 0.129; *p* = 0.006) and (aggression)^2^ that only explained a 1.7% variation in the aggression score. Whereas, in a race relationship with (aggression)^2^, Malay and Chinese races were significantly associated and explained 4.3% variance of aggression score. Students who commonly experienced hunger showed a significant linear association (*β* = 0.130; *p* = 0.013) with (aggression)^2^. Significant associations were also evident in frequent junk food intake, including desserts (*β* = 0.167; *p* < 0.001), carbonated drinks (*β* = 0.115; *p* = 0.013), and any fast-food consumption (*β* = 0.110–0.159; *p* = 0.001–0.037). Contrarily, zero intake of vegetables was significantly (*β* = 0.124; *p* = 0.023) associated with (aggression)^2^. In summary, most of the biological factors explained only 0.01 to 4.2% of the variation in the (aggression score)^2^.

**Table 1:** Association between biological factors and (adolescent aggression)^2^ by simple linear regression (*N* = 463).

Variables	(Adolescent aggression)^2^			Value of *p*
	Simple linear regression			
	*r* ^2^	*β*	*F*	
Sex (*N* = 463)				
Female (*N* = 244)	0.017		7.763	<0.001^*^
Male (*N* = 219)		0.129		0.006^*^
Race (*N* = 463)				
Malay^a^ (*N* = 349)	0.043		6.831	<0.001^*^
Chinese (*N* = 79)		−0.180		<0.001^*^
Indian (*N* = 24)		−0.097		0.035
Others (*N* = 11)		0.054		0.243
Head Injury (*N* = 460)				
No^a^ (*N* = 32)	0.001		0.692	<0.001
Yes (*N* = 428)		0.039		0.406
Hungry (*N* = 462)				
Never^a^ (*N* = 135)	0.014		2.193	<0.001^*^
Sometimes (*N* = 249)		0.048		0.364
Often (*N* = 60)		0.130		0.013^*^
Very often (*N* = 18)		0.043		0.371
Water intake (*N* = 463)				
7 or more times per week^a^ (*N* = 7)	0.008		1.186	<0.001
4–6 times per week (*N* = 38)		−0.021		0.651
1–3 times per week (*N* = 69)		−0.003		0.943
0 times per week (*N* = 349)		−0.086		0.065
Diary product intake (*N* = 461)				
0 times per week^a^ (*N* = 89)	0.010		1.502	<0.001
1–3 times per week (*N* = 243)		−0.111		0.070
4–6 times per week (*N* = 83)		−0.043		0.457
7 or more times per week (*N* = 46)		0.002		0.973
Diary product intake (*N* = 461)				
0 times per week^a^ (*N* = 89)	0.010		1.502	<0.001
1–3 times per week (*N* = 243)		−0.111		0.070
4–6 times per week (*N* = 83)		−0.043		0.457
7 or more times per week (*N* = 46)		0.002		0.973
Desserts (*N* = 462)				
0 times per week^a^ (*N* = 38)	0.042		6.634	<0.001^*^
1–3 times per week (*N* = 287)		0.018		0.827
4 or more times per week (*N* = 137)		0.167		<0.001^*^
Carbonated drinks (*N* = 462)				
0 times per week^a^ (*N* = 115)	0.013		1.938	<0.001^*^
1–3 times per week (*N* = 287)		0.080		0.134
4 or more times per week (*N* = 60)		0.115		0.013^*^
Fast food (*N* = 462)				
0 times per week^a^ (*N* = 117)	0.031		4.930	<0.001^*^
1–3 times per week (*N* = 287)		0.110		0.037^*^
4 or more times per week (*N* = 58)		0.159		0.001^*^
Fruits (*N* = 463)				
7 or more times per week^a^ (*N* = 48)	0.008		1.188	<0.001
4–6 times per week (*N* = 126)		−0.016		0.829
1–3 times per week (*N* = 231)		−0.107		0.178
0 times per week (*N* = 58)		−0.035		0.587
Vegetables (*N* = 462)				
7 or more times per week^a^ (*N* = 107)	0.012		1.873	<0.001^*^
4–6 times per week (*N* = 122)		0.034		0.560
1–3 times per week (*N* = 172)		0.033		0.574
0 times per week (*N* = 61)		0.124		0.023^*^

a Reference group.

^*^Significant.

All psychological factors were significantly associated with (aggression)^2^ except for emotional intelligence (*β* = −0.22; *p* = 0.637) as described in Table 2. The results of Pearson’s correlation showed a significant moderate positive correlation between attitude toward aggression (*r* = 0.314, *p* < 0.001) and (aggression)^2^. The simple linear regression test presented a significant and linear association of attitude toward aggression (*β* = 0.314; *p* < 0.001), normative beliefs regarding aggression (*β* = 0.270; *p* < 0.001), personality trait (*β* = 0.272; *p* < 0.001), and the (aggression)^2^, which explained the 9.9, 7.3, and 7.4% of the aggression score variation, respectively.

**Table 2:** Association between psychological factors and (adolescent aggression)^2^ by Pearson’s correlation and simple linear regression.

Variables	(Adolescent aggression)^2^				
	Correlation	Simple linear regression			Value of *p*
	Pearson’s correlation	*r* ^2^	*β*	*F*	
Attitude toward aggression (*N* = 455)	0.314	0.099	0.314	49.586	<0.001^*^
Normative Beliefs about aggression (*N* = 456)	0.270	0.073	0.270	35.737	<0.001^*^
Personality trait (*N* = 454)	0.272	0.074	0.272	36.234	<0.001^*^
Emotional Intelligence (*N* = 446)	−0.022	0.001	−0.022	0.223	0.637

*Significant.

The results obtained from the correlation analysis and simple linear regression test between social factors and (aggression)^2^ are illustrated in Table 3. There was a weak, significant negative correlation between family income and (aggression score)^2^ (*r* = −0.123, *p* < 0.05). This showed that low-level family income is associated with higher levels of aggression. There was a significant moderate positive correlation between peer deviant affiliation and (aggression score)^2^ (*r* = 0.386, *p* < 0.001). As for exposure due to domestic violence, the relationship was significant but weak (*r* = 0.222, *p* < 0.001). Other significant social factors associated with (aggression)^2^ observed were smoking (*r*^2^ = 5.6%, *p* < 0.001) and drug abuse (*r*^2^ = 0.9%, *p* = 0.036).

**Table 3:** Association between social factors and (adolescent aggression)^2^ by Pearson’s correlation and simple linear regression.

**Variables**	(Adolescent Aggression)^2^				
	Correlation	Simple linear regression			Value of *p*
	Pearson’s correlation	*r* ^2^	*β*	*F*	
Log_10_(family income) (*N* = 319)	−0.123	0.015	−0.123	4.869	0.028^*^
Family structure (*N* = 459)					
Both parents^a^ (*N* = 396)		0.005		1.235	<0.001
Other caregivers (*N* = 15)			−0.019		0.680
Single parents (*N* = 48)			0.069		0.137
Family environment (*N* = 450)	−0.080	0.006	−0.080	2.876	0.091
Log_10_(Peer deviant affiliation) (*N* = 462)	0.386	0.149	0.386	80.365	<0.001^*^
Log_10_(Domestic violence) (*N* = 449)	0.222	0.049	0.222	23.092	<0.001^*^
Smoking					
No^a^ (*N* = 435)		0.056		27.168	<0.001^*^
Yes (*N* = 27)			0.236		<0.001^*^
Alcohol					
No^a^ (*N* = 424)		0.004		1.703	<0.001
Yes (*N* = 38)			−0.061		0.193
Drug abuse					
No^a^ (*N* = 448)		0.009		4.365	<0.001^*^
Yes (*N* = 9)			0.097		0.037^*^

a Reference group.

*Significant.

### Multivariate analysis

A total of 15 significant independent variables from a simple linear regression test (*p* < 0.05) were included in the multivariate analysis (37) to determine predictors of aggression in this study. A standard multivariate linear regression test was performed to establish the predictive model for adolescent aggression. Over and above the aforementioned, the linearity of the relationship of the model, independence of error and outcome, constant variance (homoscedasticity), and normally distributed error based on the residual analysis were also met. No multicollinearity was identified as evidenced by a tolerance value of more than 0.10 and Variance Inflation.

Factor (VIF) of <10. Outliers were checked by inspecting the Mahalanobis distances that were produced by the multivariate analysis.

A final BACKWARD model was selected, which best explained (aggression)^2^ with the highest number of significant predictors. A significant regression equation was found (*F*(8,286) = 15.98, *p* < 0.001), which accounted for 29% of the variation in (aggression)^2^. The final model indicates that the combination of selected independent variables significantly (*p* < 0.001) predicts adolescent aggression. The prediction equation was: (Aggression)^2^ = 0.871 + [(−0.291)(Log_10_Family Income)] + 0.208 (Malay race) + 0.345 (desserts intake 4 times and more per week) + 0.089 (attitude) + [2.183 (Log_10_Peer deviant affiliation)].

Five independent variables resulted in a unique and statistically significant prediction of adolescent aggression from the final model as shown in Table 4: low family income, Malay race, dessert intake 4 or more times per week, attitude toward aggression, and peer deviant affiliation. Family income was the only variable that significantly and negatively predicted adolescent aggression (*β* = −0.106; *p* = 0.034). A deficit of RM1 of the total household monthly income leads to a 0.291, 95% CI [−0.560, −0.022] increase in total aggression score. In the Malay race (*β* = 0.101; *p* = 0.048), a total aggression score was predicted as 0.208, 95% CI [0.002, 0.414]; for desserts intake of four and more times per week (*β* = 0.162; *p* = 0.001) it was predicted as 0.345, 95% CI [0.135, 0.555]; and for attitude toward aggression (*β* = 0.247; *p* < 0.001), it was predicted as 0.089, 95% CI [0.053, 0.125]. The predictor that mostly predicts adolescent aggression was peer deviant affiliation (*β* = 0.264; *p* < 0.001), as the one total score increment will further increase the total aggression score by 2.183, 95% CI [1.337, 3.028].

**Table 4:** Multivariate linear regression analysis summary for predictors of (adolescent aggression)^2^ (*N* = 463).

**Variable**	***Β***	***β***	**95% CI**	***t*-test**	**Value of *p***
Malay race	0.208	0.101	0.002–0.414	1.986	0.048^*^
Desserts intake 4 and more times per week	0.345	0.162	0.135–0.555	3.229	0.001^*^
Attitude toward aggression	0.089	0.247	0.053–0.125	4.827	<0.001^*^
Log_10_(family income)	−0.291	−0.106	−0.560 to −0.022	−2.132	0.034^*^
Log_10_(peer deviant affiliation)	2.183	0.264	1.337–3.028	5.080	<0.001^*^

*Significant.

## Discussion

The median total aggression score obtained from this study was categorized as low among public secondary school students in the district. Direct comparison with other research findings is limited to different scale measurements and study populations. Findings among high-risk youth aged 15–40 years old in Klang Valley (15) and public secondary schools in Selangor (13) showed a moderate level of aggression. Notwithstanding these differences in aggression score findings, the prevalence trend of crime and bullying cases among secondary school students in Hulu Langat is still on the increase (38), with the highest number of crime cases being among students aged 16–18 years old (8).

Biological predictors of adolescent aggression examined in this study were Malay race and frequent dessert intake. Most of the Malay population in this study (75.4%) contributed to aggressive behavior among school students, similar to bullying findings obtained among public school students in Kuala Lumpur, Malaysia by Ismail et al. in 2014. Aggression among adolescents of major racial groups can be a result of different racial statuses and power influences on school students’ behavior (39). Major ethnic groups have no difficulty exercising their power over minorities (40).

Poor nutrition and low-quality diets have been shown to predict bullying among youth (16). This includes high sugar consumption that had strong and consistent relationships with risk behaviors, including physical fighting and bullying among adolescents (41). This could be explained by an increase in insulin production from a high dietary sugar intake, which resulted in behavioral changes such as poor self-control (42) due to reactive hypoglycaemia and poor glucose tolerance. Furthermore, adolescents’ dietary practices on snack and confectionery consumption were high (89.5–98.8%) as obtained from a dietary survey among Singaporean and Malaysian adolescents (43).

The only psychological predictor obtained from this study was the attitude toward aggression. High attitude scores among the students showed their positive evaluation to perform aggressive behavior, regardless of the impact on oneself and another person. This finding was also evident among school students in Selangor (13). There are several possible explanations relating to this prediction but was mainly influenced by the students’ environment. A similar positive attitude toward aggression among family and friends in a hostile environment resulted in this attitude among themselves (13). They may become desensitized to aggressive behavior (44) and also experience a maladaptive emotional regulation process similar to victimization (45). Another factor that can influence this positive attitude toward aggression is violence reported in the media (46) and exposure of students to video games, movies, and current online content. However, this factor was not investigated in this study as it was not highly statistically significant as compared to other factors.

As for social predictors of adolescent aggression, lower family income and peer deviant affiliation were identified. Lower family income was associated with adolescent aggression in both developed (47) and developing (48) countries. Unsatisfactory living conditions and interest in material possessions may influence such behavior, as materialism was negatively associated with gratitude and happiness (49). Materialistic individuals also rely on extrinsic sources for their fulfillment, resulting in low self-esteem (50). This may trigger an aggressive reaction toward other people, as they become threatened when being marginalized due to their low socioeconomic status (44).

Peer pressure is an important influence on the development of aggression among adolescents as they naturally tend to choose peers with similar characteristics (51). Peer attachment and peer deviant affiliation were found to significantly predicted aggression among secondary school students in previous researches (13, 48). Deviant peers offer adolescents opportunities to engage in antisocial behaviors by having similar attitudes, rationalization, and motivation to support such behavior (52).

Other important factors of adolescent aggression were significant in the bivariate analysis of this study, such as normative beliefs regarding aggression and family environment. For school students, normative belief can be influenced by expectations and perceptions from friends in the school environment (53). Family environment, including family conflict, family cohesion, family members’ expressiveness, and achievement orientation significantly predicted adolescents’ delinquency behavior (14). Therefore, although these factors were statistically insignificant in the multivariate analyses, their contribution to adolescent aggression may prove to be significant to public health, due to interrelations with other predictors and their detrimental impact on society.

The study conformed to the biopsychosocial theory of adolescent aggression as significant factors from each biological, psychological, and social domain were elicited. It also highlighted some important biological aspects like nutritional factors in adolescent aggression development. Despite that, the present study has some limitations. This study is cross-sectional, which limits the temporal causality effect of predictors toward adolescent aggression. The self-reported measurements among school students may also provide biased information in terms of response, such as family income. Therefore, the data extracted were reliant on the respondents’ accuracy, truthfulness, and perception. Another limitation of this study was the unfeasibility to investigate the early life factors of adolescent aggression, such as birth complications and nutritional deficiency in the early years of child development.

Recommendations on future adolescent aggression studies can be conducted with several options of different study designs and methodologies. Longitudinal cohort study allows the temporal causality investigation of factors contributing to adolescent aggression development, including the early biological life factors. In terms of the explanation of these factors, triangulation with other methods of data collection, such as in-depth interviews with students and parents and data documentation on aggressive related disciplinary problems among the school student can also be applied in the Mixed Methods study design. Based on this, certain information biases could be reduced, such as having information on total family income directly from parents rather than from the students.

As for service recommendations, the findings in this study can provide information for more focused and targeted interventional strategies to curb aggression among adolescents based on the significant predictors. As for target population intervention, the focus should be on the school area of the Malay majority who come from low socioeconomic status, as adolescents from this background are at a higher risk of becoming aggressive. They need to be educated on the possible causes and negative impacts of aggression and how to respond and communicate well with others, so they can develop negative attitudes toward aggression. This effort should start before they reach early adulthood when behavior is established and therefore becomes more difficult to change.

The school nutrition policy also needs to be highlighted and enforced at the school canteen as frequent dessert intake can influence aggressive manifestations among adolescents. The detrimental health and behavioral effects of high sugar intake among school students need to be conveyed to all stakeholders including parents, teachers, and policymakers. Strategies to promote a supportive school environment, educate the students on how to cope with peer pressure, peer support, and team assistance should be present in school so that students can have a positive influence on socialization. Parent involvement to guide and monitor their children’s activities should also be encouraged, which in turn will assist with the adolescents’ journey toward independence.

Therefore, overcoming aggression among adolescents requires collaboration by multiple stakeholders on various contributing factors. Adolescents are the future generation and will be the decision-makers whom the country will depend on.

## Conclusion

Adolescent aggression determinants include important biological, psychological, and social predictors, which collectively contribute to aggressive manifestations. These are Malay ethnicity, nutritional deficiency, attitude toward aggression, low family income, and peer deviant affiliation.

## Data availability statement

The raw data supporting the conclusions of this article will be made available by the authors, without undue reservation.

## Ethics statement

The studies involving human participants were reviewed and approved by Ethics Committee for Research Involving Human Subject of Universiti Putra Malaysia” (JKEUPM Ref No: JKEUPM-2018-228). Written informed consent to participate in this study was provided by the participants' legal guardian/next of kin.

## Author contributions

FF has made a substantial contribution to the concept of the article, including the acquisition, analysis, interpretation of data, and writing the article. NZ and AB revised the article critically for important intellectual content. All authors contributed to the article and approved the submitted version.

## Funding

A University Putra Malaysia Research Grant (Geran Inisiatif Putra Siswazah; GP-IPS/2018/9640800) was used by the researcher to cover expenses relating to this study execution.

## Conflict of interest

The authors declare that the research was conducted in the absence of any commercial or financial relationships that could be construed as a potential conflict of interest.

## Publisher’s note

All claims expressed in this article are solely those of the authors and do not necessarily represent those of their affiliated organizations, or those of the publisher, the editors and the reviewers. Any product that may be evaluated in this article, or claim that may be made by its manufacturer, is not guaranteed or endorsed by the publisher.
